# Genetics for population and public health

**DOI:** 10.1093/ije/dyx008

**Published:** 2017-02-15

**Authors:** John L Hopper

**Affiliations:** Centre for Epidemiology and Biostatistics, School of Population and Global Health, University of Melbourne, Carlton, VIC 3010, Australia.

Epidemiologists and public health practitioners are somewhat wary of genetics, and for good reason. The historical catch phrases ‘nature versus nurture’, or ‘genes versus environment’, have created a culture clash. These simplistic views are built around an inherent assumption of genetic determinism, and the belief that the causes of diseases can be partitioned into two or three groups: genetic, environmental and perhaps ‘stochastic’^1^ (which, to my mind, can’t be genetic and just represents lack of knowledge about the environment). When it comes to public or population health, this thinking hasn’t translated into anything useful except fuel for debates.

## Heritability

For a continuous trait, the concept of ‘heritability’—or more specifically ‘genetic variance’—was, in effect, defined by Ronald Aylmer Fisher while in his 20s in a seminal paper published nearly a century ago.^2^ As well as reconciling Mendelian inheritance for categorical traits with causes of variation across a fixed population for continuous traits, Fisher made several novel statistical advances in regression and analysis of variance. He also provided an elegant demonstration that height is highly heritable, in that it had a very large component of additive genetic variance in proportion to total cross-sectional variance for a given population.

But cause of variation is not the same as cause per se. For example, there have been clear increases in average height across recent generations around the world, especially in rapidly developing countries, which reflect changes in (early life) environment, not genes. Fisher^3^ was dismissive of heritability, because the information about genetic variance was ‘largely jettisoned’ when ‘only reported as a ratio to this hotch-potch of a denominator’ (the total variance), which depends on the population in question, let alone how one models the mean.^4^

So, whereas knowing which animal and plant characteristics have high heritability informs breeding within a fixed environment, it doesn’t necessarily have utility for public health. On the other hand, finding that a trait has little or no (genetic) heritability, despite being correlated in relatives, does direct public health thinking to focus on environmental factors shared by relatives (see Hopper and Mathews^5^).

When it comes to heritability of disease (a binary trait), the concept is flawed.^6^ It is not the ‘proportion of disease caused by genetic factors’, as even prominent geneticists assume.^7^ Instead, it involves an imaginary concept (liability), and the estimate of heritability depends on a specific model of risk (normal distribution; all-or-nothing risk about a threshold) for which distributional and risk assumptions can never be tested, and different assumptions lead to different estimates^6^—hardly a solid basis for a scientific paradigm.

Heritability estimates tell us nothing about the limitations of the environment on influencing a trait, as exemplified in the height example above. Heritability estimates also do not tell us the limitation of genetics in influencing a trait, which is naturally given by the disease distribution for monozygotic (genetically identical) twin pairs: see below.

As for ‘missing heritability’,^8^ it is not clear from the literature whether the denominator is based on the familial aggregation for genetically identical pairs or on some other estimate of what can be achieved by genetics. And the role of non-genetic factors in explaining familial aggregation^9^ is a topic rarely broached in the genetics literature. Perhaps the quest to discover ‘missing heritability’ would be more fruitful if it was broadened to finding ‘missing familiality’.

## Effects of the environment and genetic risk gradient

On the other hand, I argue that genetics provides a tremendously important source of information that can be used by epidemiologists and others to improve the health of the population. Phenylketonuria is a poster-book example; a heel prick taken at birth can be used to detect a genetic disorder that can be readily treated by diet.^10^

From the epidemiological and public health perspective, a key issue is how well a risk factor differentiates cases from controls within a given population. This can be determined from the risk gradient, expressed for example in terms of the change in odds per standard deviation of the risk factor, adjusted for age and other risk factors, in the population about which inference is being made (OPERA).^11^

The maximum risk gradient that can be caused by genetic factors can be inferred from the disease distribution of genetically identical twin pairs. Importantly, and not necessarily well recognized, this underlying risk gradient must be substantial even for diseases for which the familial risk (increased risk of having the disease if you have an affected relative) would naively be interpreted as modest.^9^^,^^12^ For example, a 4-fold increased risk for the co-twin of an affected genetically identical twin, or a 2-fold familial risk ratio for having disease in a first-degree relative (typical of many common diseases), imply that the interquartile risk ratio for the underlying familial causes must be 20-fold or more (equivalent to an odds ratio per standard deviation of > 3 and an area under the receiver operator curve of ∼ 0.8). Finding all the familial causes, therefore, would have a profound influence on risk prediction.

The inference above is based on the multiplicative model that has been the basis of most epidemiological risk analyses, and this model now appears to give a good approximation to the way known independent genetic markers across the genome are associated with disease (see e.g. Mavaddat *et al.*^13^).

**Figure 1:**
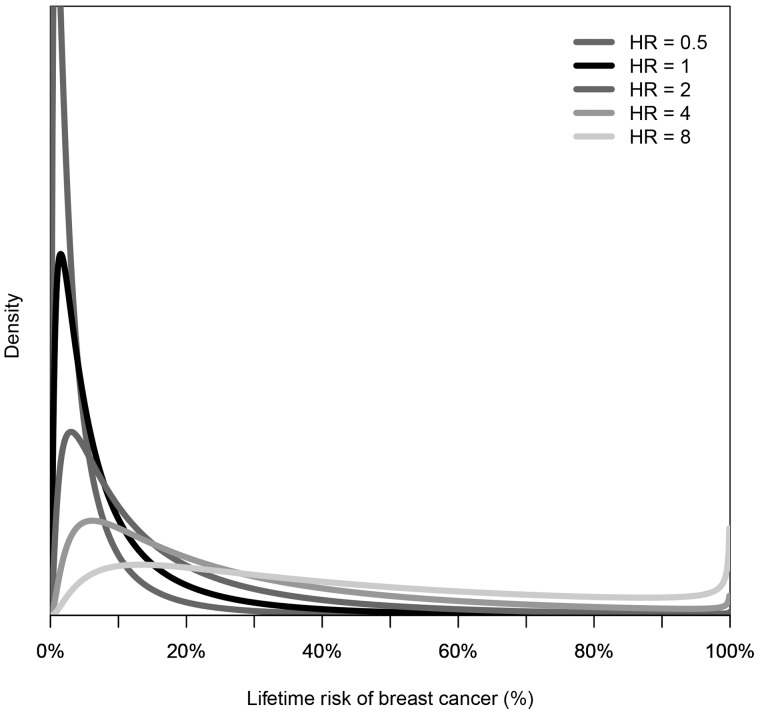
Distribution of lifetime risk of breast cancer for women with different hazard ratios (HRs) compared with the population, derived under a mixed major polygenic risk model, as described in Dowty *et al.*^18^ for breast cancer with a familial relative risk (for first-degree relatives) of 2.

One consequence of the multiplicative model is that the risk distribution is log normal and therefore has a long tail. The risk for the vast majority of people is well below the population ‘average’. The risk distribution for a group of people selected for being at increased risk based on one or more risk factors is not the same as the risk for the population shifted to the right, let alone (log) normally distributed. Instead, it is more like a uniform distribution with wide variance, as illustrated in Figure 1 for breast cancer.

Therefore, a factor that changes risk by a few-fold has little relevance for the vast majority of people. But if its risk gradient is independent of genetic/familial risk, that factor could be associated with a substantial change in absolute risk for a non-trivial proportion.

**Figure 2:**
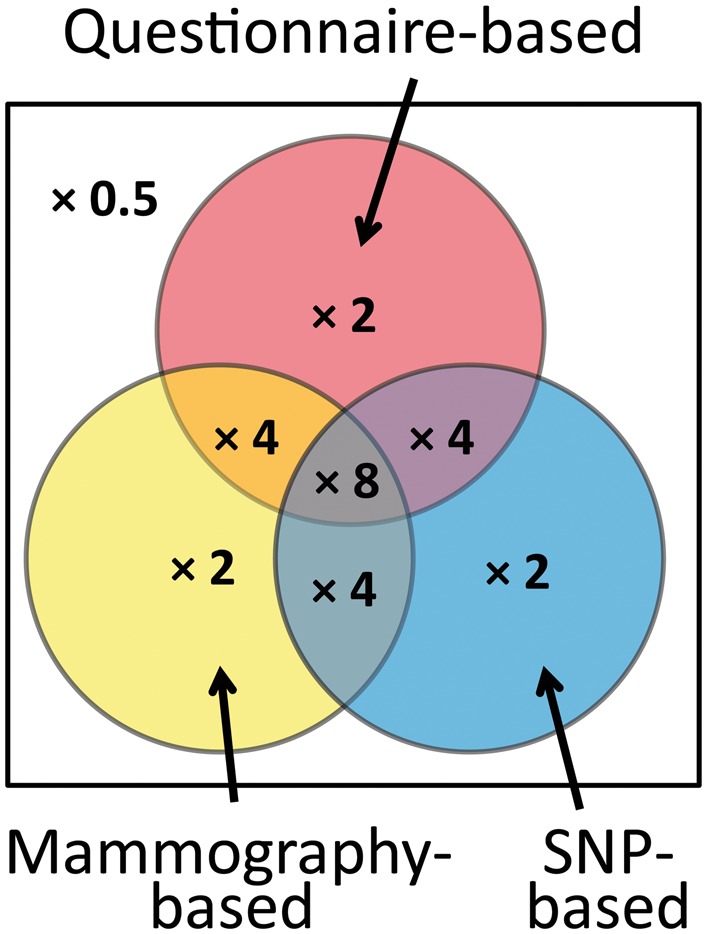
Categories of women in the top 25% of risk factors based on questionnaires (including multigenerational family history), mammographic density measures and genetic risk scores based on single nucleotide polymorphisms (SNPs). Numbers represent the incidence for that group compared with the general population.

Suppose a risk factor has a risk gradient of OPERA = 1.5, as in breast cancer is the case for: (i) the latest single nucleotide polymorphism (SNP)-based genetic risk scores; (ii) mammographic density measures of risk;^14^ and (iii) risk scores based on multigenerational family history;^15^ see Figure 2. As a group, women in the top 25% of the risk distribution for these factors have about twice the average incidence for the population. Their risk distribution is shown in Figure 1 for a hazard ratio (HR) of 2. These risk factors are at most weakly correlated with one another.^16^ Therefore, women in the top 25% of the risk distribution for two of these factors could have about four times average incidence (see Figure 1 for HR = 4). Those in the top 25% of all three risk factor distributions could have about eight times average incidence, similar to carriers of *BRCA1* and *BRCA2* mutations (see Figure 1 for HR = 8). About 1.5% of the population will be in the top 25% of all three factors, whereas only 0.7% of the population are thought to be mutation carriers.^17^

Therefore, judicious use of mammographic images and multigenerational family history data followed by relatively cheap targeted testing for a SNP-based risk score could identify a larger group of women at the same high risk as carriers much more readily than would gene panel testing even if extended to the population. Just as importantly for screening, about 40% of women who are not in the top 25% for any of these factors might be at half the population risk, and most will be at very small risk (see Figure 1 for HR = 0.5)

But here’s the rub. Knowing that you are at high risk for a disease is not necessarily helpful unless there are known, proven and acceptable ways of mitigating that risk. People at different levels of genetic risk might differ in susceptibility to a given risk factor, or even be susceptible to different risk factors. For example, a rising incidence and younger age at diagnosis of type 1 diabetes in recent decades has been observed for people with lower risk HLA class II genotypes, but not for those with the highest risk genotype for whom the incidence and age at diagnosis has been constant.^18^ There is indirect evidence that, for carriers of mutations in the DNA mismatch repair genes (Lynch syndrome), the effect of familial modifiers on their risk of colorectal cancer is more important than it is for the general population^18^ and could involve different genes.

Therefore, considering gene–gene and gene–environment interactions is important. If gene–environment interactions on the multiplicative scale do not exist, they will exist on the additive scale and vice versa, and these two scenarios have different implications. Epidemiological studies need also to be conducted on people at high or increased genetic risk, as well as for people across the full spectrum of genetic risk.^19^^,^^20^ Genetics could play a substantive role in precision prevention and screening if it is incorporated with other risk factors. In this and many other ways, epidemiologists should own genetics and use it to serve public health.

## Funding

This work was supported by a Senior Principal Research Fellowship from The National Health and Medical Research Council of Australia (Grant #1023434).
